# Validation of the extended thrombolysis in cerebral infarction score in a real world cohort

**DOI:** 10.1371/journal.pone.0210334

**Published:** 2019-01-10

**Authors:** Daniel Behme, Ioannis Tsogkas, Ruben Colla, Roland G. Gera, Katharina Schregel, Amélie C. Hesse, Ilko L. Maier, Jan Liman, David S. Liebeskind, Marios-Nikos Psychogios

**Affiliations:** 1 Department of Neuroradiology, University Medical Center Göttingen, Göttingen, Germany; 2 Department of Medical Statistics, University Medical Center Göttingen, Göttingen, Germany; 3 Department of Neurology, University Medical Center Göttingen, Göttingen, Germany; 4 Neurovascular Imaging Research Core and Stroke Center, Department of Neurology, UCLA, Los Angeles, CA, United States of America; University of Münster, GERMANY

## Abstract

**Background:**

A thrombolysis in cerebral infarction (TICI) score of 2b is defined as a good recanalization result although the reperfusion may only cover 50% of the affected territory. An additional mTICI2c category was introduced to further differentiate between mTICI scores. Despite the new mTICI2c category, mTICI2b still covers a range of 50–90% reperfusion which might be too imprecise to predict neurological improvement after therapy.

**Aim:**

To compare the 7-point “expanded TICI” (eTICI) scale with the traditional mTICI in regard to predict functional independence at 90 days.

**Methods:**

Retrospective review of 225 patients with large artery occlusion. Angiograms were graded by 2 readers according the 7-point eTICI score (0% = eTICI0; reduced clot = eTICI1; 1–49% = eTICI2a, 50–66% = eTICI2b50; 67–89% = eTICI2b67, 90–99% = eTICI2c and complete reperfusion = eTICI3) and the conventional mTICI score. The ability of e- and mTICI to predict favorable outcome at 90days was compared.

**Results:**

Given the ROC analysis eTICI was the better predictor of favorable outcome (p-value 0.047). Additionally, eTICI scores 2b50, 2b67 and 2c (former mTICI2b) were significantly superior at predicting the probability of a favorable outcome at 90 days after endovascular therapy with a p-value of 0.033 (probabilities of 17% for mTICI2b50, 24% for mTICI2b67 and 54% for mTICI2c vs. 36% for mTICI2b).

**Conclusions:**

The 7-point eTICI allows for a more accurate outcome prediction compared to the mTICI score because it refines the broad range of former mTICI2b results.

## Introduction

Endovascular therapy (EVT) has become the standard of care in the treatment of large artery occlusion (LAO) in the anterior circulation[1]. A major reason for the success of EVT is the high rate of successful recanalization which was achieved in several recent trials[1]. To reach this point, a parallel development of materials and techniques has been going on for more than a decade resulting in higher recanalization results and faster interventions[2]. To categorize the success of the interventions, several grading systems have been used. Historically, the thrombolysis in myocardial infarction (TIMI) score was the first grading system used in EVT[3]. Later, the thrombolysis in cerebral infarction (TICI) score was modified leading to the currently most popular grading system mTICI[4]. At the same time there was a significant change in the definition on what should be considered a successful recanalization result raising the threshold from TICI2a to TICI2b more recently[5]. However; mTICI2b is an imprecise grade covering 50–99% of reperfusion and several studies have shown that patients with complete (mTICI3) or near complete reperfusion results had significantly better clinical outcome compared to patients with mTICI2b results[6, 7]. More recently Tung et al. proposed TICI2c as an additional category in the TICI scoring system reasoning that TICI2c reperfusion results in significantly better early neurological improvement[8]. In clinical practice, an improvement of the angiographic result from TICI2b to TICI2c is feasible and safe in a relevant number of cases[6]. However; even if a TICI2c category is applied mTICI2b still covers a reperfused territory spectrum of 51–89% and whether it correlates with different clinical outcomes is still questionable. Liebeskind et al. therefore reanalyzed the HERMES data using a novel 7-point TICI score (eTICI) in regard to predict functional outcome at 90days and found significant differences between TICI2a/TICI2b50 and TICI2b50 and TICI2b67 suggesting the superiority of the eTICI score in a large study cohort[9].

### Aims

To examine the ability of the novel expanded 7-point TICI (eTICI)[9, 10] score compared to the conventional mTICI score regarding the prediction of favorable functional outcome at 90days in a real word setting with a focus on the novel categories replacing TICI2b.

## Methods

### Study population

We screened our prospectively acquired stroke database for all patients who underwent EVT for large artery (carotid T, middle cerebral artery (MCA), basilar artery (BA) and posterior cerebral artery (PCA) occlusion between January 2015 and April 2017. No other in- or exclusion criteria were applied yielding a total number of 225 patients. The need for an informed study consent was waived due to the retrospective nature of this study. In the context of the required emergency treatment consent was obtained whenever possible for mechanical thrombectomy. Data were derived from a prospectively acquired database, which is approved by the local ethics committee (IRB University Medical Center Goettingen 13/7/15An).

### Endovascular treatment

Within 6 hours of symptom onset patients were eligible for EVT if a large artery occlusion was present and an intracranial hemorrhage was ruled out by either multi detector (MDCT/MDCTA) or flat panel computed tomography/angiography (FDCT/FDCTA)[11, 12]. Patients were treated regardless of their initial Alberta Stroke Program Early CT Score (ASPECTS) within the first 6 hours of symptom onset. Beyond 6 hours from symptom onset patients received additional computed tomography perfusion (CTP) imaging and only patients with a cerebral blood volume (CBV)-CTP ASPECTS score of 5 or higher were treated. Full dose intravenous tissue plasminogen activator (tPA) was administered within 4.5 hours from symptom onset whenever indicated according to the current guidelines in Germany. All patients who were shipped to our department from a primary stroke center for EVT received MDCT/MDCTA or FDCT/FDCTA imaging before EVT to rule out interim hemorrhage and to verify the vessel occlusion. Endovascular treatment was performed under conscious sedation in the majority of patients; general anesthesia was only applied in cases with lack of consciousness to protect airways or in cases with severe agitation. In the majority of cases a triaxial approach using an 8F femoral long sheath (Destination Terumo Medical, Somerset, NJ,USA) or an 8F guiding catheter (VISTA Brite tip, Cordis, Milpitas, CA, USA) a 5 or 6F intermediate catheter (Catalyst, Stryker Kalamazoo, USA, Penumbra ACE 64/68, Penumbra, Alameida, CA, USA), a mircocatheter (Trevo pro 14/18, Stryker) and a stent-retriever (Trevo pro Vue 4x30mm, Stryker) was deployed. Thrombectomy was carried out using a variety of techniques within the study period including a direct aspiration first pass (ADAPT), Solumbra[13] and stent assisted vacuum locked extraction (SAVE)[14, 15]. From July 2015 to the end of the study period all patients were treated using the SAVE technique that has been described in detail before. The number of passes were on the discretion of the treating physician. I.a. tPA was not used in any case.

### Image analysis

**Fig 1:**
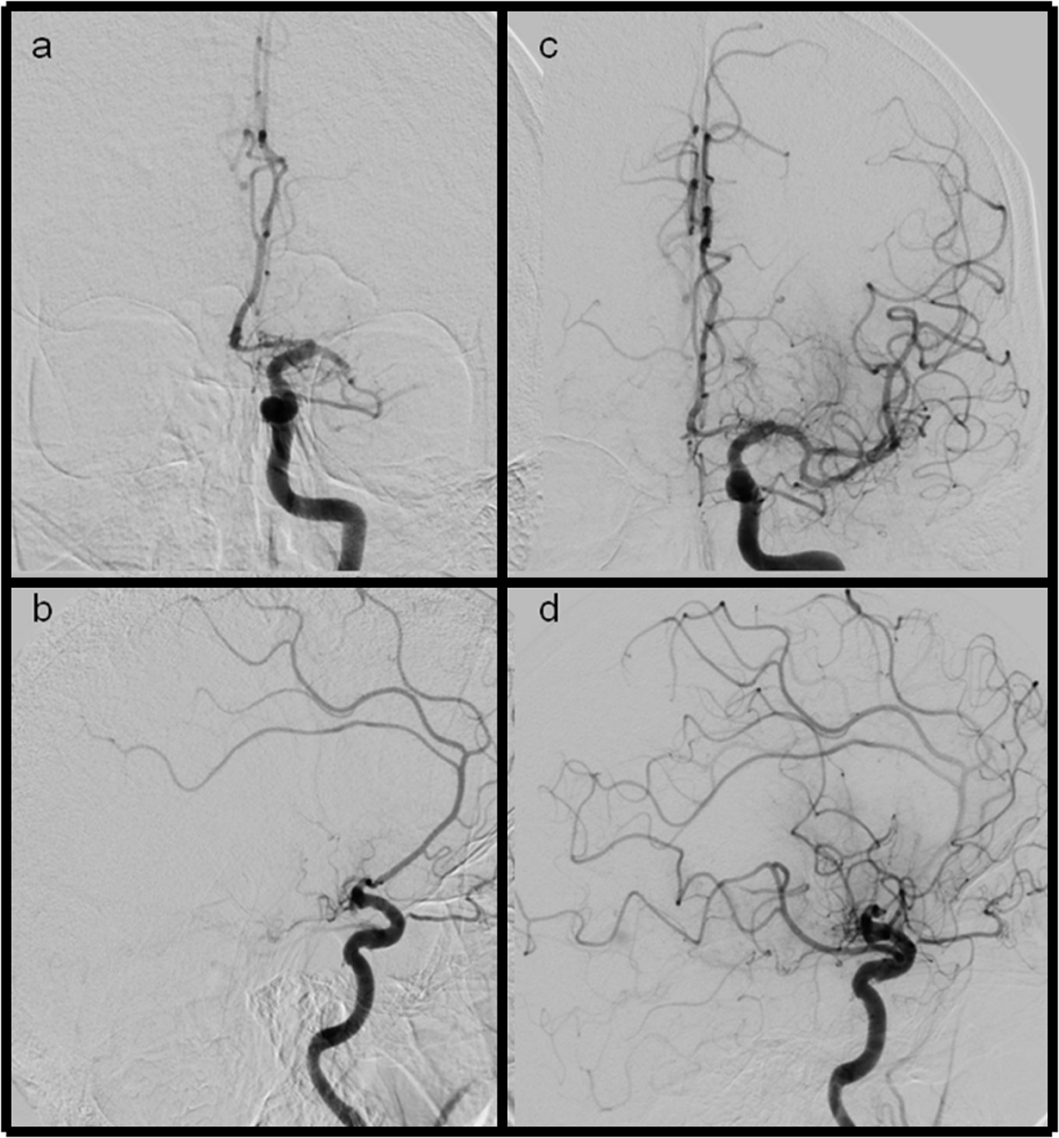
**Ap and lateral views of LAO and eTICI2b50 (mTICI2b) reperfusion results** (a+b) ap and lateral views showing a M1 occlusion of the left side (c+d) after recanalization the inferior MCA trunk remains occluded, additionally the frontal division is not fully reperfused. Severe filling defects can be seen in the parietal and frontal part of the MCA territory.

**Fig 2:**
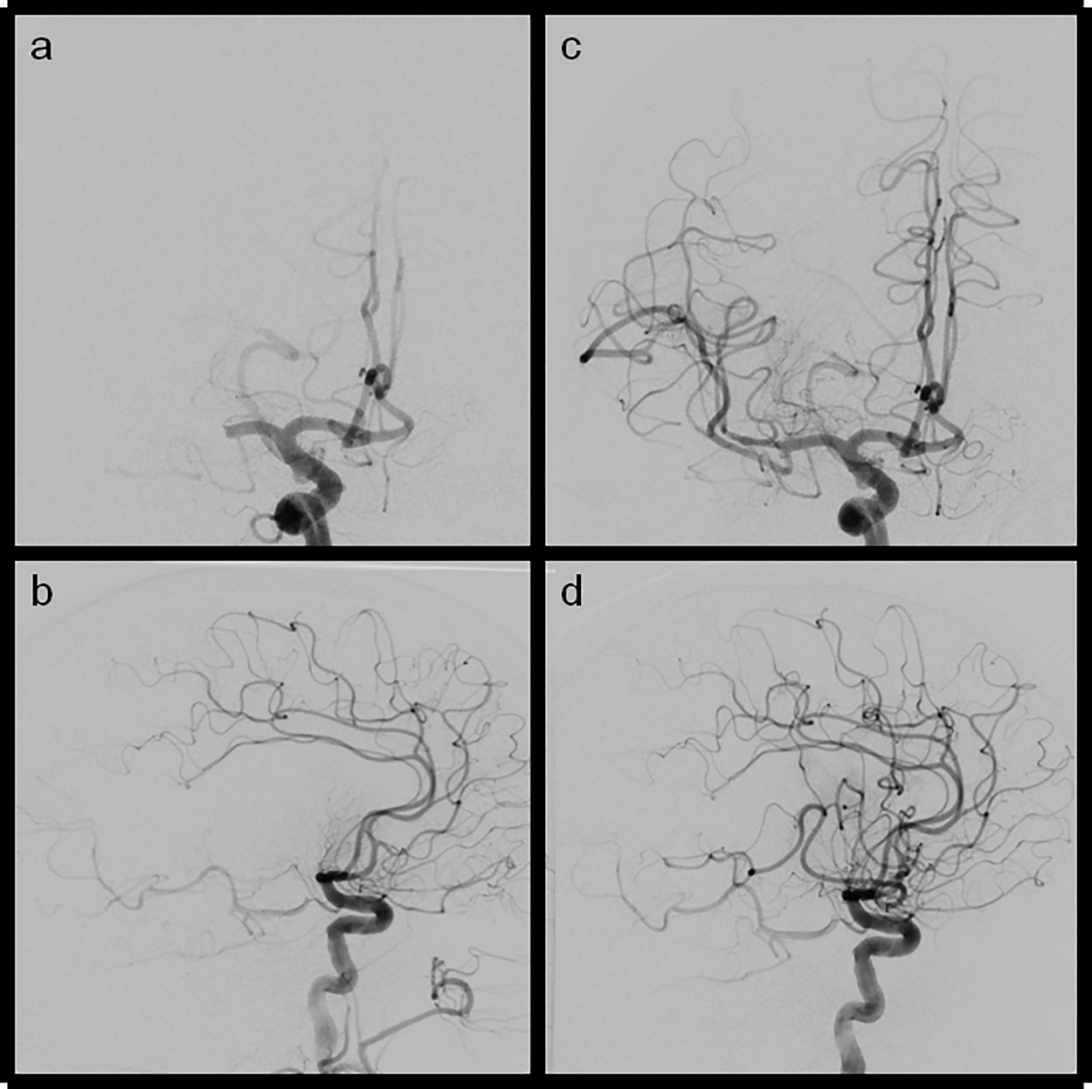
**Ap and lateral views of LAO and eTICI2b67(mTICI2b) reperfusion results** (a+b) ap and lateral views of a M1 occlusion of the right side (c+d) ap and lateral view after reperfusion showing a remaining MCA M2 division being still occluded which is led to incomplete reperfusion (central and parietal MCA territory not fully reperfused).

**Fig 3:**
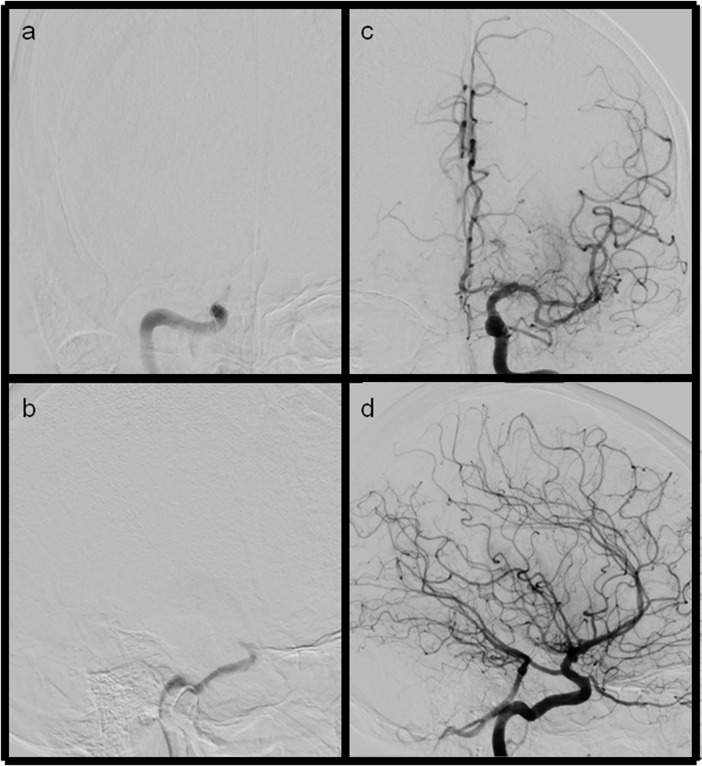
**Ap and lateral views of LAO and eTICI2c (mTICI2b) reperfusion results** (a+b) ap and lateral views showing a distal ICA occlusion (c+d) after thrombectomy only very small distal branches in parietal location do not fill.

Two readers with different experience (I.-T. >5 years and R.-C. > 3 years) in interventional neuroradiology evaluated the angiograms blinded to all clinical data. The percentage of reperfusion was estimated based on anterior-posterior (a.p.) and lateral view images of the final angiograms according the definitions of the eTICI score. All images were already judged for their mTICI score directly after the intervention and before the data was entered into our data-base. The 7-point scale of eTICI is as follows: eTICI0 = 0% = mTICI0; eTICI 1 = minimal flow past the occlusion but no perfusion = mTICI1; eTICI2a = 1–49% = mTICI2a; eTICI2b50 = 50–66% = mTICI2b; eTICI2b67 = 67–89% = mTICI2b; eTICI2c = 90–99% = mTICI2c; eTICI3 = complete reperfusion = mTICI3. Examples of the reperfusion grading (mTICI2b divided into eTICI2b50, 2b67 and -2c) are given in Figs 1–3. In cases of discrepancies the images were re-evaluated in a joint session until a consensus was reached which then was used for further analysis.

The original rating was used for calculation of the interclass correlation coefficient. For the posterior circulation the assessment was adjusted to the relevant downstream territory and judged accordingly.

### Neurological assessment

The following data were taken from our prospective stroke data-base: age; sex; symptom onset/wake up; site of occlusion; baseline National Institute of Health Stroke Scale (NIHSS), baseline modified Rankin Scale (mRS), discharge NIHSS, discharge mRS; 90days mRS; all intra hospital time points: CT; tPA start; groin puncture; reperfusion and final angiogram. All neurological assessment were performed and recorded in our data-base by experienced vascular neurologists (IL.M. >5 years and J.L. >10 years). 90 days follow-up was obtained during a routine visit of the patients in our neuro-vascular outpatient clinic by the same vascular neurologists; both were not blinded to the angiographic and previous clinical data.

### Statistics

Statistical analyses were performed using SAS 9.4 (SAS, Wallisellen, CH) or Med Calc (Med Calc, Ostend, Belgium).

At the start of the investigation population characteristics were inspected. An overview of the results of that investigation can be found in Table 1.

**Table 1:** Patients characteristics.

Item	Median (IQR) or n (%)
Age	76 (66–82)
Initial NIHSS	16 (10–20)
Male sex	109 (48%)
**Site of occlusion**	
ICA	64 (28.4%)
MCA	132 (58.7%)
Tandem	44 (19.6%)
BA	24 (10.7%)
PCA	5 (2%)
**Procedural information**	
Groin to recanalization time, min	50 (32–74)
eTICI0 (mTICI0)	9 (4%)
eTICI1 (mTICI1)	4 (1.8%)
eTICI2a (mTICI2a)	22 (9.8%)
eTICI2b50 (mTICI2b)	23 (10.2%)
eTICI2b67 (mTICI2b)	71 (31.6%)
eTICI2c (mTICI2b)	46 (20.4%)
eTICI3 (mTICI3)	50 (22.2%)
**Neurological outcome at 90days**	
mRS0	26 (12%)
mRS1	32 (14%)
mRS2	30 (13%)
mRS3	27 (12%)
mRS4	31 (14%)
mRS5	22 (10%)
mRS6	57 (25%)

Agreement between both raters was calculated using Cohen’s Kappa and interclass correlation coefficient (ICC).

All analysis comparing mTICI and eTICI were performed only on those patients who presented with large artery occlusion of the anterior circulation. For the direct comparison of mTICI2b and eTICI2b50, -2b67 and -2c results of all patients were analyzed. Curves for the Receiver Operating Characteristics (ROC) of eTICI and mTICI were calculated using good outcome (mRS 0–2 at 90 days) versus unfavorable outcome (mRS 3–6 at 90 days) as dichotomized classification variables. The difference between both ROC-curves was then further investigated using the DeLong test comparing their areas under the curves (AUC)[16]

To further investigate if a granulation of mTICI2b into corresponding eTICI2b50, -2b67 and -2c scores yielded further information, testing the differences in outcome of these specific groups was done using the Jonckheere-Terpstra test.

To control for the family-wise error rate we used Bonferroni correction for the p-value.

## Results

A total of 225 patients were included in the study. Median age was 76 (IQR: 66–82) years, the NIHSS at baseline was 16 (IQR: 10–20) 48% were male. Site of occlusion were predominantly the MCA (133/225, 58.7%) and the ICA (64/225, 28.4%), in 44 cases (44/225, 19.6%) a tandem occlusion was present. In a minority of cases the posterior circulation was affected; in 28 cases (28/255, 10.7%) a BA occlusion was treated and in 5 cases (5/255, 0.02%) a PCA occlusion. The median time from puncture to recanalization was 50 min (IQR: 32–74). Recanalization was not achieved in 13 cases (13/225, 5.2%), accounting for 9 cases of eTICI0 (9/255, 3.5%) and 4 cases of eTICI1 (4/255, 1.6%). In another 22 cases (22/225, 8.7%) an eTICI2a result was achieved. In the remaining 84.4% of the cases a substantial perfusion was restored with eTICI2b50 (23/225, 10.2%); eTICI2b67 (71/225 31.6%), eTICI2c (46/225, 20.4%) and eTICI3 (complete recanalization) (50/225, 22.2%). A favorable outcome (mRS ≤ 0–2 at 90days) was achieved in 88 cases (88/225, 39.1%). For a summary of all patient characteristics see Table 1.

Overall agreement (Cohens Kappa) between raters was 0.706 (95% CI: 0.645–0.769). Interclass correlation (ICC) within eTICI was 0.862 (95%CI: 0.821–0.894).

**Fig 4:**
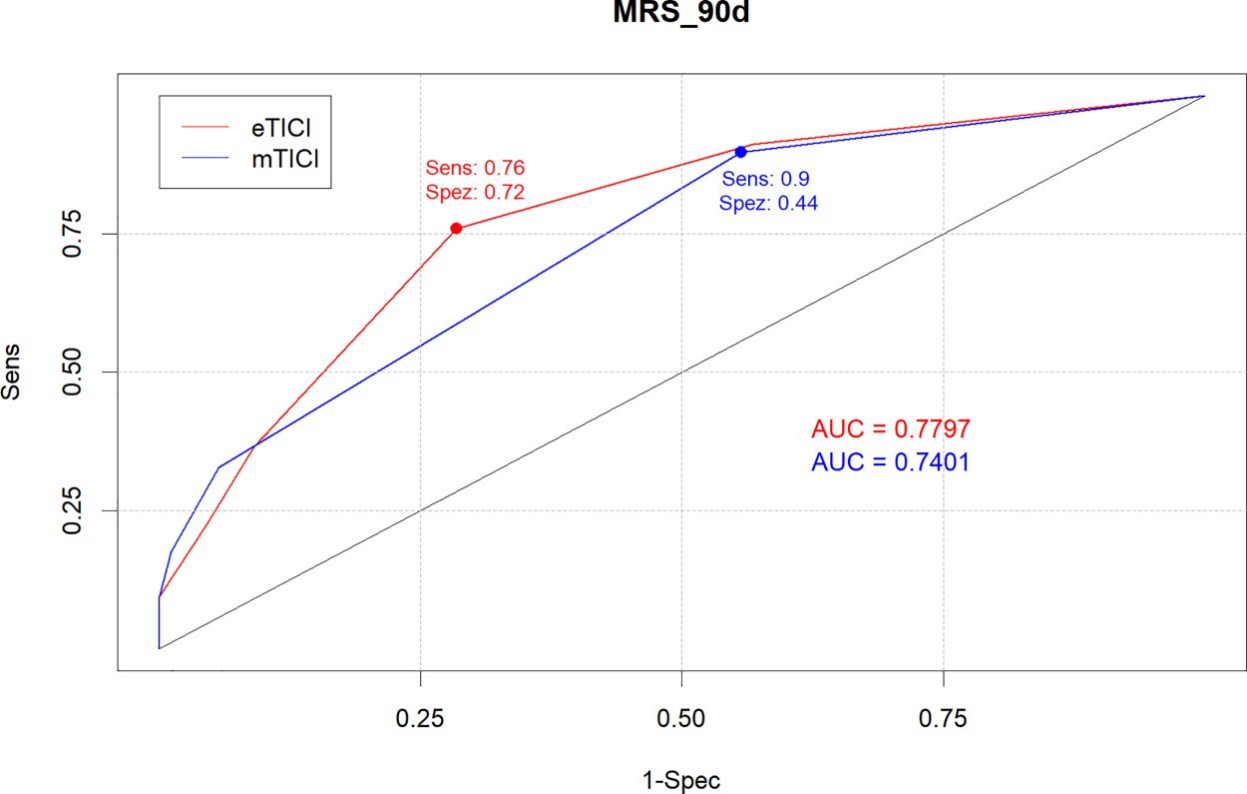
ROC for eTICI and mTICI.

For the analysis comparing eTICI scores with mTICI scores, ROC analysis with dichotomized mRS scores at 90 days (mRS 0–2) as outcome were calculated using the respective scores. Derived from the ROC-curve the AUC could be calculated leading to an AUC of 0.779 (95%CI: 0.719–0.839) for eTICI and 0.740 (95%CI: 0.683–0.797) for mTICI. Using the Youden-Index as criteria for optimal cutoff values, optimal cutoff for eTICI was achieved at eTICI2b67 with 75.9% sensitivity (95%CI: 67.86%-82.8%) and a 71.6% specificity (95%CI: 60.98%-80.7%). For mTICI we obtained a cutoff at mTICI2b with 89.7% sensitivity (95%CI: 83.44%-94.3%) and 44.3% specificity (95%CI: 33.72%-55.3%) (Fig 4). Comparing AUC of both raters resulted in a p-Value of 0.047.

Analyzing outcome differences of eTICI2b50, -2b67 and -2c scores on patients who were simultaneously categorized as mTICI2b resulted in a significant association between rising scores and rising success probability (p = 0.033). In our cohort mTICI2b patients had a probability of 36% for a good outcome, while the differentiated success probabilities were 17% for eTICI2b50; 24% for eTICI2b67 and 54% for eTICI2c (Table 2).

**Table 2:** Probability of good outcome at 90 days according to mTICI2b/eTICI 3–5.

	mRS = 0,1,2	mRS = 3,4,5,6	%(mRS = 0,1,2)
**mTICI2b**	44	78	36,06%
**eTICI2b50**	4	19	17,39%
**eTICI2b67**	17	54	23,94%
**eTICI2c**	25	21	54,34%

## Discussion

This is the first study evaluating the novel 7-point expanded TICI (eTICI) score in a real world cohort [9, 10]. Our results show that an expansion of the widely used and accepted mTICI score leads to several improvements. First of all the eTICI leads to a better overall outcome prediction as validated by ROC analysis. Second of all outcome stratification of eTICI2b50, 2b67 and 2c compared to mTICI2b resulted in more accurate prediction of outcome at 90days. In our cohort a significant trend was observed between eTICI2b50 to 2c results and the probability of good outcome with higher eTICI scores underline the importance of a more sophisticated interpretation of angiographic result after EVT. These findings confirm what has been reported by Liebeskind et al. when applying the eTICI score to the HERMES data. However in our study we could not find a significant difference between eTICI2b50 and eTICI2b67 regarding the prediction of functional outcome at day 90, although there was a trend towards better outcomes in the eTICI2b67 group. Additionally angiographic results in our cohort in general were substantially better compared to what was found in the analysis of the HERMES data which might reflect the advances in thrombectomy techniques over the past years [9, 17]. The novel score is very easy to implement and does require only moderate changes in the daily routine of angiographic grading. However; we would recommend using the term “thrombectomy”- rather than thrombolysis in cerebral infarction score. eTICI expands the concept proposed by Tung et al. or Alemekhlafi et al. recently[8, 18]. Both working groups proposed an additional category within the mTICI system which does reflect a near complete reperfusion result, the so called mTICI2c outcome. Tung et al. found significant differences between early and 90 day clinical outcome when comparing patients with mTICI2c with patients experiencing mTICI2b results. Interestingly, this doesn’t hold true when comparing mTICI3 reperfused patients with mTICI2c reperfused patients[8]. Almekhlafi likewise demonstrated that mTICI2c resulted in better outcomes when compared to mTICI2b results[18]. However; a severe limitation of mTICI2c is its missing clear definition as it has been originally defined as near complete reperfusion. Volny et al. reported low or moderate inter-rater agreements when angiographic results were graded according mTICI including a mTICI2c category by stroke physicians or neuroradiologist and only when graded in a consensus reading an excellent inter-rater agreement was observed[19]. Regarding this limitation, eTICI does offer a clear percentage cutoff values to evaluate the grade of reperfusion which may help for a more accurate and coherent grading. Compared to an overall Cohens Kappa of 0.609 and an ICC of 0.845 that were reached by the readers of the Tung group for mTICI grading, we calculated an overall Kappa of 0.706 and an ICC of 0.862, which is comparable. Scoring clear percentage-cutoffs may be difficult in several cases but as we could show in this study a distinction between three different mTICI2b grades seems to be feasible. In this context, one should be aware of the fact that oTICI other than mTICI defined a successful reperfusion result as >2/3 filling of the affected territory[4]. Considering that eTICI provides a clearer definition on what others may refer to as mTICI2c, eTICI does offer a solution that incorporates both grading systems in one (oTICI and mTICI). The fundamental idea of a more sophisticated and more precise grading system is the basis of all the aforementioned studies. This idea simply derives from the fact that a grading system in which everything between 51 and 99% (or to near complete) reperfusion is called a “good” result is insufficient in terms of outcome prediction. In this study, 36% of patients with an mTICI2b classification had a favorable outcome at 90 days, but when partitioned into three eTICI groups, the difference of good outcome probability became evident. On the upper end, patients with an eTICI2c reperfusion result had a favorable outcome in 54% of the cases whereas on the lower end only 17% of patients had a favorable outcome at 90 days when an eTICI2b50 result was achieved.

Considering the results of Tung et al. achieving a near complete reperfusion may be as good as complete reperfusion[8]. Therefore, it may be worth trying to improve angiographic results in cases which initially ended with a relatively bad mTICI2b result. Kasemacher et al. recently reported similar results to Tung et al. in regards to the probability of good outcome when various reperfusion grades were compared. In their study with 246 patients the rate of good functional outcome at 90 days was significantly different between mTICI2b and mTICI2c/3 results (28.7% vs. 46.5%, p = 0.008)[6]. Even more interestingly Kaesmacher et al. reviewed the angiographic records for procedures in which an mTICI2b result could be improved to mTICI2c/3. They found 28 patients in which they improved the angiographic result and consecutively the clinical result as this did not differ between the groups of primary or secondary mTICI2c/3 results. Taking this into account, a more advanced TICI score could guide the interventionalist towards a refined decision making in the future.

There are several limitations to this study. First the retrospective evaluation of prospectively acquired data from a single center. The angiographic grading was blinded to the clinical outcome. However, the neurological assessment was not blinded to the angiographic result. Unlike others, we included not only cases with anterior circulation occlusion because in our mind all cases need a reperfusion grading and we performed the grading according the percentage definitions of eTICI regarding the downstream territory distal to the initial occlusion site. Obviously mTICI was not designed for the posterior circulation which might impact our results. In this context it has been shown that TICI scoring is associated with poor inter-rater agreement[20] when used for the posterior circulation which certainly has an impact on our results although the number of cases with posterior circulation stroke in this study was low. In this regard it is important to notice that the regression analysis were only performed for patients with anterior circulation stroke. Finally, our reperfusion results and procedural times improved over the study period which may affect the results in terms of exact time points on which a certain eTICI result corresponds to a certain clinical outcome.

## Conclusions

The eTICI score allows for an overall better outcome prediction compared to mTICI. Thereby eTICI2b50, 2b67 and 2c allow for a more precise outcome prediction compared to mTICI2b and may help to refine clinical decision making in the future.

## Abbreviations

(EVT): Endovascular therapy
(eTICI): Expanded thrombolysis in cerebral infarction
(mTICI): Modified thrombolysis in cerebral infarction
(LAO): Large artery occlusion
